# Microbially mediated sulfur oxidation coupled with arsenate reduction within oligotrophic mining–impacted habitats

**DOI:** 10.1093/ismejo/wrae110

**Published:** 2024-06-20

**Authors:** Xiaoxu Sun, Qizhi Chen, Max M Häggblom, Guoqiang Liu, Tianle Kong, Duanyi Huang, Zhenyu Chen, Fangbai Li, Baoqin Li, Weimin Sun

**Affiliations:** National-Regional Joint Engineering Research Center for Soil Pollution Control and Remediation in South China, Guangdong Key Laboratory of Integrated Agro-environmental Pollution Control and Management, Institute of Eco-environmental and Soil Sciences, Guangdong Academy of Sciences, Guangzhou 510650, China; Guangdong-Hong Kong-Macao Joint Laboratory for Environmental Pollution and Control, Guangzhou Institute of Geochemistry, Chinese Academy of Sciences, Guangzhou 510640, China; National-Regional Joint Engineering Research Center for Soil Pollution Control and Remediation in South China, Guangdong Key Laboratory of Integrated Agro-environmental Pollution Control and Management, Institute of Eco-environmental and Soil Sciences, Guangdong Academy of Sciences, Guangzhou 510650, China; Guangdong Key Laboratory of Environmental Pollution and Health, School of Environment, Jinan University, Guangzhou 511443, China; Department of Biochemistry and Microbiology, Rutgers University, New Brunswick, NJ 08901, United States; Guangdong Key Laboratory of Environmental Pollution and Health, School of Environment, Jinan University, Guangzhou 511443, China; National-Regional Joint Engineering Research Center for Soil Pollution Control and Remediation in South China, Guangdong Key Laboratory of Integrated Agro-environmental Pollution Control and Management, Institute of Eco-environmental and Soil Sciences, Guangdong Academy of Sciences, Guangzhou 510650, China; College of Environmental Science and Engineering, Donghua University, Shanghai 201620, China; National-Regional Joint Engineering Research Center for Soil Pollution Control and Remediation in South China, Guangdong Key Laboratory of Integrated Agro-environmental Pollution Control and Management, Institute of Eco-environmental and Soil Sciences, Guangdong Academy of Sciences, Guangzhou 510650, China; College of Environmental Science and Engineering, Hunan University, Changsha 410082, China; National-Regional Joint Engineering Research Center for Soil Pollution Control and Remediation in South China, Guangdong Key Laboratory of Integrated Agro-environmental Pollution Control and Management, Institute of Eco-environmental and Soil Sciences, Guangdong Academy of Sciences, Guangzhou 510650, China; School of Environment, Key Laboratory of Yellow River and Huai River Water Environment and Pollution Control, Ministry of Education, Henan Normal University, Xinxiang 453007, China; National-Regional Joint Engineering Research Center for Soil Pollution Control and Remediation in South China, Guangdong Key Laboratory of Integrated Agro-environmental Pollution Control and Management, Institute of Eco-environmental and Soil Sciences, Guangdong Academy of Sciences, Guangzhou 510650, China; Guangdong-Hong Kong-Macao Joint Laboratory for Environmental Pollution and Control, Guangzhou Institute of Geochemistry, Chinese Academy of Sciences, Guangzhou 510640, China; National-Regional Joint Engineering Research Center for Soil Pollution Control and Remediation in South China, Guangdong Key Laboratory of Integrated Agro-environmental Pollution Control and Management, Institute of Eco-environmental and Soil Sciences, Guangdong Academy of Sciences, Guangzhou 510650, China; Guangdong-Hong Kong-Macao Joint Laboratory for Environmental Pollution and Control, Guangzhou Institute of Geochemistry, Chinese Academy of Sciences, Guangzhou 510640, China; National-Regional Joint Engineering Research Center for Soil Pollution Control and Remediation in South China, Guangdong Key Laboratory of Integrated Agro-environmental Pollution Control and Management, Institute of Eco-environmental and Soil Sciences, Guangdong Academy of Sciences, Guangzhou 510650, China; Guangdong-Hong Kong-Macao Joint Laboratory for Environmental Pollution and Control, Guangzhou Institute of Geochemistry, Chinese Academy of Sciences, Guangzhou 510640, China

**Keywords:** sulfur oxidation, arsenate reduction, stable isotope probing, genome mining

## Abstract

Arsenate [As(V)] reduction is a major cause of arsenic (As) release from soils, which threatens more than 200 million people worldwide. While heterotrophic As(V) reduction has been investigated extensively, the mechanism of chemolithotrophic As(V) reduction is less studied. Since As is frequently found as a sulfidic mineral in the environment, microbial mediated sulfur oxidation coupled to As(V) reduction (SOAsR), a chemolithotrophic process, may be more favorable in sites impacted by oligotrophic mining (e.g. As-contaminated mine tailings). While SOAsR is thermodynamically favorable, knowledge regarding this biogeochemical process is still limited. The current study suggested that SOAsR was a more prevalent process than heterotrophic As(V) reduction in oligotrophic sites, such as mine tailings. The water-soluble reduced sulfur concentration was predicted to be one of the major geochemical parameters that had a substantial impact on SOAsR potentials. A combination of DNA stable isotope probing and metagenome binning revealed members of the genera *Sulfuricella*, *Ramlibacter*, and *Sulfuritalea* as sulfur oxidizing As(V)-reducing bacteria (SOAsRB) in mine tailings. Genome mining further expanded the list of potential SOAsRB to diverse phylogenetic lineages such as members associated with *Burkholderiaceae* and *Rhodocyclaceae*. Metagenome analysis using multiple tailing samples across southern China confirmed that the putative SOAsRB were the dominant As(V) reducers in these sites. Together, the current findings expand our knowledge regarding the chemolithotrophic As(V) reduction process, which may be harnessed to facilitate future remediation practices in mine tailings.

## Introduction

Arsenic (As) is the 20th most abundant element in the Earth’s crust and a ubiquitously distributed primary contaminant [1]. Naturally occurring As, primarily in the form of sulfidic ores, is frequently associated with diverse metal deposits, such as Cu, Ag, Zn, Pb, Fe, and Sb [2]. Release of As from solid materials (e.g. soils, sediments, and bedrock) to aquatic environments is caused by either natural geochemical processes or anthropogenic activities [3–6]. Metal mining is one of the dominant anthropogenic causes for continental erosion and produces an extraordinary quantity of wastes, which are referred to as mine tailings [7, 8]. Metal(oid) contaminants, including As, released from mining activities are a serious environmental threat to the surrounding region and its residents [9, 10] and have attracted extensive attention regarding possible prerequisites for remediation [9, 10].

Microbial reduction of arsenate [As (V)] to arsenite [As(III)] may significantly enhance the toxicity and mobility of As, and is considered one of the key processes that causes As release from contaminated soils and tailings [11, 12]. There are two known pathways for biological As(V) reduction. The *ars* operon encodes the detoxification As(V) reduction pathway [13]. The *ars* pathway mediates uptake of dissolved As(V) and reduces it to As(III) in the cytoplasm, which makes it less relevant to release of solid-phase As in the environment [14]. In contrast, dissimilatory As(V) reduction, which is mediated by a periplasmic dissimilatory As(V) reductase encoded by the *arrAB* genes, reduces As(V) and releases As(III) to the extracellular environment [15]. Accordingly, the dissimilatory As(V) reduction pathway, which uses both soluble and insoluble As(V), may be more relevant to As release from contaminated sites than the detoxification As(V) reduction pathway, which requires soluble As(V) [16, 17].

Microbial dissimilatory As(V) reduction depends on coupling with the oxidation of an electron donor. Various organic substrates (lactate, acetate, formate, and aromatic compounds) have been shown to mediate the dissimilatory As(V) reduction process [18]. The availability of organic substrates, however, is often limited in oligotrophic As-contaminated sites (i.e. mine tailings), and chemolithoautotrophs using inorganic electron donors, particularly sulfur compounds, dominate in such environments [19, 20]. Thus, sulfur oxidation–mediated As(V) reduction (SOAsR) is proposed to be a potentially important biogeochemical process in oligotrophic As-contaminated habitats. Indeed, the high energy yield generated during biological sulfur oxidation is comparable to the energy yield of organic substrate oxidation during the As(V) reduction process [21]. SOAsR was first observed in Mono Lake, California, United States, a closed-basin lake with active microbial sulfur and arsenic cycles [22]. Strain MLMS-1 (a previously unclassified *Desulfobacterota* that is closely related to the family *Desulfurivibrionaceae*) isolated from this lake was responsible for SOAsR [22]. In addition to MLMS-1, the halophilic strain *Halarsenatibacter silvermanii* SLAS-1, isolated from Searles Lake, CA, USA, was reported to perform SOAsR under salt-saturated conditions [23].

Mine tailings are oligotrophic As-contaminated habitats with high concentrations of sulfur but low total organic carbon contents [20]. Therefore, the geochemical conditions in mine tailings are likely to favor the biogeochemical process of SOAsR. The presence and contribution of SOAsR in mine tailings, however, remain elusive and require further investigation to increase understanding of the release of As from mining activities to the environment. In this study, multiple mine tailings from southern China were selected to determine the potential for SOAsR. A combination of geochemical experiments, molecular techniques, and bioinformatic analyses was employed to elucidate the microbial mechanisms of SOAsR and reveal the significance of SOAsR in mine tailing environments. In addition, environmental metagenome analysis was performed to quantify the relative abundance of putative sulfur-oxidizing As(V)–reducing bacteria (SOAsRB) in mine tailing sites. The aims of the current study were as follows: (i) demonstrate the presence and geochemical controls of SOAsRB in oligotrophic As-contaminated mine tailing habitats, (ii) identify and characterize SOAsRB, and (iii) investigate the contribution of SOAsRB to As(V) reduction in mine tailings.

## Material and methods

### Site description and sample collection

Multiple tailing samples were investigated, including two different tailing samples from the Fankou Pb/Zn mine (FK and FK2); individual samples from one Pb/Zn/Sb mine tailing in Nandan, Guangxi (CWSK); one Sb mine tailing in Huangjia, Hunan (HJ); one Sb mine tailing in Lizhixixiang, Hunan (LZXX); one Sb mine tailing in Qinglong, Guizhou (QL); one As mine tailing in Shimen, Hunan (SM); one Pb/Zn mine tailing in Tangxi, Hunan (TXX); and one Sb mine tailing in Xikuangshan, Hunan (XKS). Bulk tailing samples were collected with a sterilized shovel and stored on ice immediately. Once transported back to the laboratory, the samples were stored at 4°C before further processing. Detailed site information and sample usage are listed in Supplementary Table S1.

### Geochemical analysis

For tailing sample characterization, the pH was measured using an HQ30d pH meter (Hach, CO, USA). The total organic carbon (TOC) and water-soluble organic carbon (WSOC) were analyzed by use of a Shimadzu TOC analyzer coupled to a solid sample module 5000A (Shimadzu, Japan). Total nitrogen, total sulfur, water-soluble SO_4_^2−^, and water-soluble reduced sulfur (WSRS) were analyzed using an ion chromatograph (Thermo Scientific, USA), which was also used to monitor changes in soluble sulfur species during incubation. Total As and Sb contents in tailings and changes in soluble As during incubation were analyzed using a liquid-chromatograph atomic fluorescence spectrometer (Haiguang, Beijing, China). More details regarding the geochemical analyses are provided in Supplementary Method 1.

### Sulfur-oxidizing As(V) reduction incubations

The activity characterization of sulfur-oxidizing As(V) reduction (SOAsR) was performed using enrichment cultures inoculated with FK tailing samples (i.e. activity analysis cultures). A total of 4 treatments (5 replicates for each treatment) were prepared for the activity incubations: (i) As+S treatment, (ii) As-only treatment, (iii) S-only treatment, and (iv) sterile As+S treatment. The source of S was 0.5 mM thiosulfate and 0.5 mM As(V) was the source of As(V). The sterilized tailing sample was prepared using gamma-radiation at 25 kGy for 10 min. For better estimation of the stoichiometry of the reaction, the As+S cultures were serially (20%) transferred twice. Additional incubations with FK tailings as inoculum were performed to compare the As(V) reduction rates under heterotrophic conditions using an organic substrate (1 mM sodium acetate) as the electron donor with rates under chemoautotrophic conditions using reduced sulfur (1 mM thiosulfate) as the electron donor. Control incubations without electron donor addition were also prepared. Details of the incubation conditions can be found in Supplementary Method 2.

The SOAsR potential and its correlation with geochemical parameters were tested using enrichment cultures (i.e. SOAsR incubation) of 6 different tailing sites (FK, FK2, CSWK, HJ, TXX, and XKS). The correlations among geochemical parameters and SOAsR potentials were analyzed by using the Pearson correlation in the corrplot package in R with a confidence level of 0.95 [24]. Triplicate samples were sacrificed for microbial community characterization at four different time points (on days 0, 10, 20, and 30).

### Stable isotope probing culture setup and quantification of functional genes

Stable isotope probing (SIP) cultures using FK tailing samples were prepared for identification of putative SOAsRB. The setup of the SIP cultures was the same as for the activity analysis cultures but with different carbon isotope substrates. Four treatments (triplicate microcosms for each treatment) were prepared [i.e. ^13^C-As+S amended with ^13^C-labeled bicarbonate, As(V), and thiosulfate; ^12^C-As+S amended with ^12^C-labeled bicarbonate, As(V), and thiosulfate; ^13^C-As amended with ^13^C-labeled bicarbonate and As(V); and ^13^C-S amended with ^13^C-labeled bicarbonate and thiosulfate]. After incubation, genomic DNA was extracted from samples by using the DNeasy Powersoil Kit (Qiagen, Dresden, Germany) following the manufacturer’s protocol. Total DNA from each sample was then separated into isopycnic fractions by ultracentrifugation according to a published protocol [25, 26]. Details of the SIP sample processing are provided in Supplementary Method 3.

The functional genes in each retrieved SIP fraction were quantified with a StepOnePlus quantitative polymerase chain reaction (Applied Biosystems, CA, USA). The Small subunit ribosomal RNA (SSU rRNA) was amplified in each DNA fraction using the bacteria-specific primer set 331F/518R [20]. The dissimilatory As(V) reductase gene *arrA* was amplified using the primer set arrA-CVF1/arrA-CVF2 [27]. The amplification conditions followed previously published protocols [28].

### DNA sequencing and analysis

The time-series samples from the SOAsR incubations and a subset of SIP fractions, based on the quantitative polymerase chain reaction results (Supplementary Table S2), were selected for amplicon sequencing. The amplicon sequencing of the V4 hypervariable region of the SSU rRNA gene was performed on an Illumina NovaSeq at Personal Biotechnology, Shanghai, China. The amplicon sequences were processed in QIIME2 [29]. The clean reads were clustered into amplicon sequencing variants (ASVs) with DADA2 [30]. The taxonomy of the ASVs were assigned against the SILVA 138 database [31].

The unfractionated total DNA from the ^13^C-As+S treatment was pooled and used for metagenomic sequencing on an Illumina NovaSeq at the Personal Biotechnology, Shanghai, China. The metagenomic reads were assembled into contigs using Metaspades [32], binned into metagenome assembled genomes (MAGs) using the Metawrap workflow [33], and dereplicated using dRep [34]. The quality of MAGs was estimated using CheckM [35]. The taxonomy of the MAGs was assigned using the Genome Taxonomy Database (GTDB) toolkit against the GTDB r207 database [36]. The functional annotation of open reading frames (ORFs) was analyzed using kofamscan against the KEGG database (Release 97.0) and the completeness of metabolic pathways were estimated with KEGGdecoder [37, 38]. Pangenomic analyses of potential SOAsRB were performed using reference genomes retrieved from GTDB r207 with pangenome workflow in Anvi’o 7.2 [39].

Phylogenetic diversity of potential SOAsRB was estimated using reference genomes from the GTDB v207 database. The *arrA*, *soxB*, and *dsrA* genes were identified using HMMER3 [40]. The functions of retrieved *dsr* genes (reductive *dsr*, reverse *dsr*, or oxidative-type *dsr*) were predicted based on the phylogenetic reconstruction. The retrieved amino acid sequences of the functional genes were aligned using Muscle and then the phylogenetic tree was constructed using RaxML [41, 42]. A total of 17 metagenomes from 7 mine tailings were employed for quantification of the abundance of SOAsRB compared to that of non–S-oxidizing As(V) reducers (Supplementary Table S1 and Table S3). The environmental tailing metagenomes from HJ, TXX, and LZXX were sequenced on an Illumina NovaSeq at Personal Biotechnology, Shanghai, China, whereas the metagenomes of CSWK, QL, SM, and XKS were sequenced previously [19, 20]. The *arrA* sequences were quantified in the tailing metagenomes using Salmon [43]. Details of sequencing analysis are provided in Supplementary Method 4.

All sequencing data generated in the current study were uploaded to the NCBI SRA database under accession number PRJNA989741.

## Results

### Geochemical analysis of the SOAsR process

**Figure 1:**
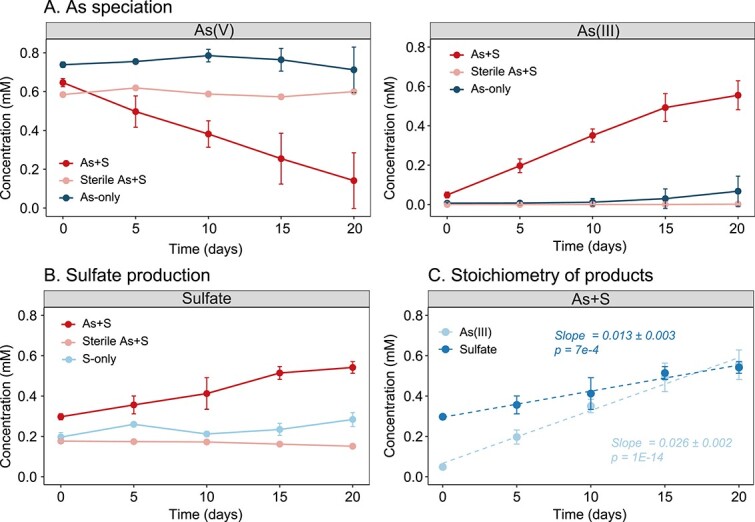
Geochemical analysis of enrichment cultures. (A) Concentration of As species; (B) SO_4_^2−^ concentration; and (C) the accumulation of products (As(III) and SO_4_^2−^) over the incubation period. Error bars indicate standard deviation of 5 replicates.


Enrichment cultures amended with both As(V) and thiosulfate (As+S treatment) indicated the presence of active microbially mediated SOAsR. Production of As(III), along with the depletion of As(V), was observed only in the As+S treatment but not in the As-only control (Fig. 1A). Moreover, the production of SO_4_^2−^ was observed in As+S treatments but not in S-only controls (Fig. 1B), suggesting that thiosulfate amendment triggered As(V) reduction. In addition, production of neither As(III) nor SO_4_^2−^ was observed in sterilized As+S controls, indicating that SOAsR was a biotic rather than an abiotic process. In the serially transferred enrichment cultures, the production rate of As(III) was 26 ± 2 μM/d, and the production rate of SO_4_^2−^ was 13 ± 3 μM/d (Fig. 1C). Accordingly, the stoichiometry of this reaction was approximately 2:1, which agreed with the predicted reaction equation:

$$ 4{\mathrm{H}\mathrm{AsO}}_4^{2-}+{\mathrm{S}}_2{\mathrm{O}}_3^{2-}+7{\mathrm{H}}_2\mathrm{O}=4{\mathrm{H}}_3{\mathrm{AsO}}_3^0+2\mathrm{S}{\mathrm{O}}_4^{2-}+6\mathrm{O}{\mathrm{H}}^{-};{\Delta \mathrm{G}}_0172\mathrm{kJ}/\mathrm{mol} $$

The ΔG_f0_ values for ${\mathrm{HAsO}}_4^{2-}$, ${\mathrm{S}}_2{\mathrm{O}}_3^{2-}$, ${\mathrm{H}}_2\mathrm{O}$, ${\mathrm{H}}_3{\mathrm{AsO}}_3^0$, $\mathrm{S}{\mathrm{O}}_4^{2-}$, and $\mathrm{O}{\mathrm{H}}^{-}$ are −714, −647, −237, −640, −745, and − 157 kJ/mol, respectively [44].

The As(V) reduction rates by the FK tailing microbiome were comparable under heterotrophic and chemoautotrophic conditions. The maximum As(V) reduction rates were 38 μM/g dry weight (dw)/d and 45 μM/g dw/d for cultures using 1 mM thiosulfate or 1 mM acetate as electron donor, respectively (Supplementary Fig. S1). Minimal As(V) reduction (1.4 μM/g dw/d) was observed in no electron donor controls.

Incubations using 6 different tailing samples were performed with both As and S additions to unveil the impact of geochemical conditions on the SOAsR process. The highest SOAsR potential, as proxied by As(V) reduction rates, was observed in the samples from CSWK tailings at 43 ± 4 μM/g dw/d, while the lowest rate was found in the samples from XKS tailings at 4.4 ± 0.1 μM/g dw/d (Fig. S2A). The Pearson correlation indicated that the SOAsR potential was positively correlated with water-soluble reduced sulfur (WSRS, *R* = 0.80, *P =* 3 × 10^−3^), total organic carbon (TOC, *R =* 0.87, *P =* 3 × 10^−5^), water-soluble organic carbon (WSOC, *R* = 0.94, *P =* 8 × 10^−7^) (Supplementary Fig. S2B), and the As concentration (*R =* 0.49, *P =* .03).

### Microbial populations responsible for SOAsR in tailing communities

The temporal shifts in microbial community compositions during SOAsR incubations were monitored through analysis of amplicon sequencing data. Decreased microbial community richness during SOAsR incubations was observed in all sites (Supplementary Fig. S3A). Furthermore, the beta diversity in Bray-Curtis distance suggested that SOAsR incubations resulted in distinct community compositions compared to the original tailing samples (Supplementary Fig. S3B). Some of the dominant ASVs in SOAsR incubations were related to the genera *Pseudomonas*, *Angustibacter*, *Stenotrophomonas*, *Thiobacillus*, and uncultured *Symbiobacteraceae* (Supplementary Fig. S3C). Some of these populations have not been previously connected with either sulfur oxidation or As(V) reduction activities.

**Figure 2:**
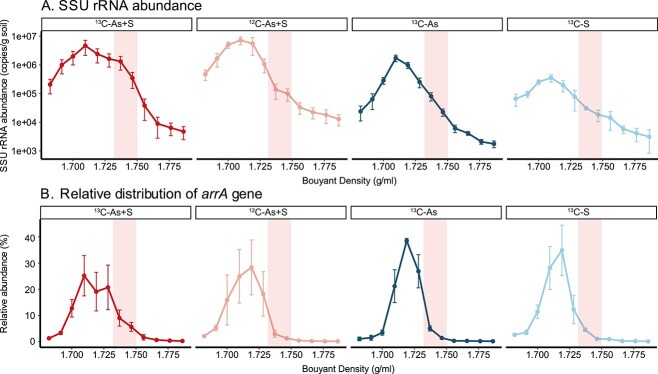
Quantification of SSU rRNA (A) and As(V) reductase gene *arrA* (B) abundances in retrieved SIP fractions. The pink shade denotes the fractions that are considered as ^13^C-incorporated heavy fractions. Error bars indicate standard deviation of triplicate samples. For the fractions that employed for sequencing analysis, please refer to Supplementary Table S2.

DNA-SIP was performed to identify the putative SOAsRB. A total of four treatments, ^13^C-As+S, ^12^C-As+S, ^13^C-As, and ^13^C-S, were established. Bicarbonate, either ^13^C-labeled or ^12^C-unlabeled, was amended as the sole carbon source for identification of chemolithoautotrophic microorganisms. DNA retrieved from SIP incubations was separated by isopycnic gradient centrifugation into heavy (buoyant density [BD] values between 1.73 and 1.75 g/ml, except for two fractions) and light (BD values between 1.70 and 1.72 g/ml) fractions based on their corresponding buoyant density (Fig. 2A and Supplementary Table S2).

The two heavy DNA fractions (as highlighted in Fig. 2A) from the ^13^C-As+S treatments accounted for 16 ± 3% of the total SSU rRNA copies of all fractions, which was significantly higher (*P =* .01) compared to the corresponding fractions in ^12^C-As+S treatments (1% ± 1% of all SSU rRNA genes), suggesting incorporation of ^13^C into biomass. Incorporation of ^13^C-labeled bicarbonate was observed in both controls as indicated by the increased relative abundance of SSU rRNA genes in heavy fractions of the controls compared to ^12^C-As+S treatments (3% ± 1% and 4% ± 0.4% for ^13^C-As and ^13^C-S, respectively). SSU rRNA gene abundance in heavy fractions of ^13^C-As+S treatments was 8 ± 4 × 10^5^ copies/g soil, compared to 1 ± 0.4 × 10^5^ copies/g soil in ^12^C-As+S treatments. The overall SSU rRNA gene copy abundance in both ^13^C-labeled and ^12^C-unlabeled As+S treatments were significantly higher than those for the two control treatments (^13^C-As and ^13^C-S). The overall SSU rRNA gene abundances were 1.4 ± 1 × 10^7^ and 2.1 ± 0.7 × 10^7^ copies/g soil in ^13^C-As+S and ^12^C-As+S treatments, respectively, while the SSU rRNA gene abundances were 3.5 ± 0.8 × 10^6^ and 1.1 ± 0.2 × 10^6^ copies/g soil in ^13^C-As and ^13^C-S controls, respectively.

The relative abundance, normalized to the total retrieved *arrA* gene copies across all fractions, of the dissimilatory As(V) reductase gene *arrA* was also enriched in the heavy fractions of ^13^C-As+S compared to other treatments (Fig. 2B). The selected two fractions of the ^13^C-As+S treatments contained roughly 15% ± 8% of the total *arrA* genes, which was significantly higher compared to the *arrA* abundance of the same fractions in ^12^C-As+S (4 ± 3%, *P = .01*), ^13^C-S (6 ± 1%, *P = .02*), and ^13^C-As (4 ± 3%, *P = .04*) treatments, respectively.

**Figure 3:**
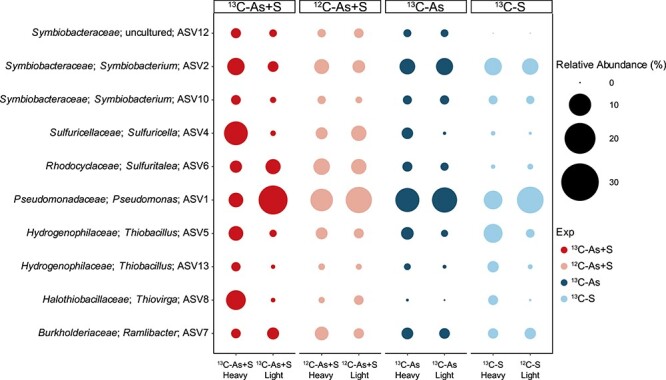
Relative abundance of dominant ASVs in SIP fractions. The size of the bubbles represents the average relative abundance of each ASV from triplicate samples, except for the heavy fraction of ^13^C-A + S samples, which was calculated from 6 replicates.

The selected heavy and light fractions (as listed in Supplementary Table S2) were subjected to amplicon sequencing for identification of putative SOAsRB. The dominant microbial ASVs in the heavy fractions of ^13^C-As+S treatments were targeted as potential SOAsRB for their ability to incorporate ^13^C-labeled bicarbonate with the amendment of both sulfur and As (Fig. 3). ASV4, belonging to the genus *Sulfuricella*, constituted 20% ± 17% of the total community in the ^13^C-As+S heavy fractions, which was significantly higher compared to all other fractions in different treatments and controls (all *P <* .001). Similarly, the *Thiovirga*-associated ASV8 was also enriched in ^13^C-As+S heavy fractions (14% ± 10%) compared to other fractions (all *P <* .001). Other ASVs showing high relative abundances in the ^13^C-As+S heavy fractions include those affiliated with the genera *Pseudomonas*, *Sulfuritalea*, and *Thiobacillus*, and the families *Comamonadaceae* and *Symbiobacteraceae*. The ASV7 was one of the dominant ASVs in the ^13^C-As+S heavy fractions and was assigned to an unclassified genus within the family *Comamonadaceae* using the Silva database. ASV7 was taxonomically related to the genus *Ramlibacter* of the family *Burkholderiaceae* (100% sequence similarity) according to the GTDB database (Supplementary Table S4) and will hereafter be referred to as a member of the genus *Ramlibacter*.

### Characterization of the functional potentials of sulfur-oxidizing As(V) reducers

Metagenomic binning was performed to recover the MAGs of putative SOAsRB for further verification of their metabolic potentials. Metagenomic binning reconstructed 60 MAGs (completeness >50% and redundancy <10%) that represented 8 microbial phyla (Supplementary Fig. S4 and Table S5), including 28 MAGs assigned to the *Pseudomonadota* (formerly *Proteobacteria*, including 24 *Gammaproteobacteria* MAGs and 3 *Alphaproteobacteria* MAGs). Several retrieved MAGs were taxonomically associated with the putative SOAsRB identified by DNA-SIP. These MAGs were related to *Sulfuricella* (MAG06, MAG54, and MAG60), *Thiovirga* (MAG41 and MAG27), *Ramlibacter* (MAG12 and MAG35), *Sulfuritalea* (MAG43 and MAG57), *Symbiobacteraceae* (MAG28,46, and 56), *Thiobacillus* (MAG14 and 19), and *Pseudomonas* (MAG42 and MAG52).

*As(V) reduction.*

**Figure 4:**
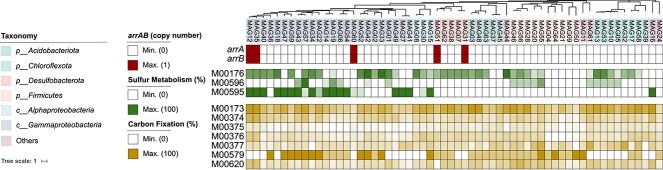
The metabolic potentials of select MAGs. The number of the *arrAB* gene copies identified in each MAG is indicated in dark red. The green heatmap indicates the completeness (%) of the sulfur metabolism pathways (M00176, assimilatory sulfate reduction; M00596, dissimilatory sulfate reduction; and M00595, thiosulfate oxidation). The yellow heatmap indicates the completeness (%) of the carbon fixation pathways (M00173, reductive citrate cycle; M00375, hydroxypropionate-hydroxybutyrate cycle; M00376, 3-hydroxypropionate bi-cycle; M00374, dicarboxylate-hydroxybutyrate cycle; M00377, reductive acetyl-CoA pathway; M00579, phosphate acetyltransferase-acetate kinase pathway; and M00620, incomplete reductive citrate cycle).

**Figure 5:**
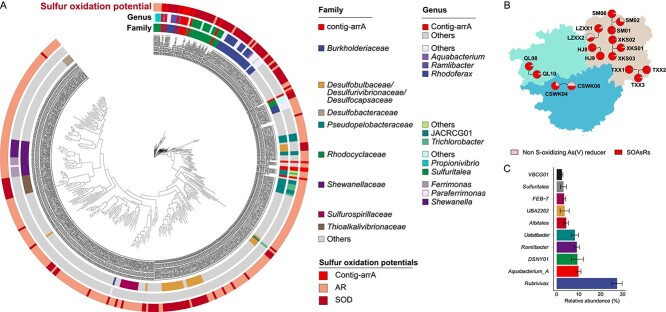
Phylogenetic diversity of putative SOAsRB and their relative abundance in tailing metagenomes. Phylogenetic reconstruction of *arrA* genes retrieved from assembled MAGs and the GTDB reference genomes (A). The relative abundance of the SOAsRB and non-S-oxidizing As(V) reducers based on the *arrA* gene (B). The top 10 most abundant SOAsRB genera identified in tailing metagenomes (C).

A total of 5 MAGs (MAG60, MAG12, MAG35, MAG51, and MAG31) encoded complete dissimilatory As(V) reductase *arrAB* genes (Fig. 4). The *arrAB* genes in these 5 MAGs were all flanked by the *arrDE* genes, which encoded a molecular chaperone TorD-like protein and a putative 4Fe-4S protein. This gene arrangement was the same as that found in the known As(V) reducing *Geobacter* sp. OR-1 [45]. Further, a conserved R/KGRY motif, which encodes for the binding site of As(V) during respiratory reduction, was also identified in all MAG-encoded *arrA* sequences [46]. Therefore, we consider that these *arr* genes encode functional respiratory As(V) reductases. While the MAGs associated with the genus *Sulfuritalea*, which was suggested as a putative SOAsRB by DNA-SIP, did not encode *arrAB* genes, 9 out of the 30 *Sulfuritalea* reference genomes encoded an *arr* gene cluster and these genes were phylogenetically related (Fig. 5A**,**Supplementary Fig. S5**,**Supplementary Table S6). Furthermore, the phylogenetic reconstruction indicated that multiple *arrA* genes identified in SIP-metagenome contigs clustered with the *arrA* sequences retrieved from *Sulfuritalea* reference genomes (Fig. 5A). Besides, other ORFs encoded in these *arrA-*containing contigs were also closely related to *Sulfuritalea* encoded sequences. This finding suggests that missing *arrA* sequences in the *Sulfuritalea*-related MAGs were caused by incomplete binning. No sequences assigned to an *arrA* gene were detected in genomes related to *Thiovirga*, *Symbiobacteraceae*, *Thiobacillaceae*, and *Pseudomonas* (data not shown). Combining the presence of an As(V) reductase *arrA* gene and the DNA-SIP results, members of the *Sulfuricella*, *Ramlibacter*, and *Sulfuritalea* were considered as As(V)-reducing bacteria and their genetic potentials of sulfur oxidation were further characterized.

*Sulfur oxidation*. Both the thiosulfate oxidation pathway (SOX, M00595) and dissimilatory sulfate reduction pathway (*dsr*, M00596) has been shown to catalyze sulfur oxidation reactions [47–49]. A total of 17 MAGs encoded a complete SOX pathway (Fig. 4). All MAGs associated with putative SOAsRB (i.e. MAGs associated with *Sulfuricella*, *Ramlibacter*, and *Sulfuritalea*) encoded complete SOX pathway genes, except for MAG60 associated with *Sulfuricellaceae*, which encoded partial SOX pathway genes. Pangenome analysis of *Sulfuricellaceae*-associated genomes (Supplementary Table S7) revealed that sulfur oxidation was a core function shared by all members of this family (Supplementary Fig. S6). Specifically, complete SOX pathway genes were identified in all *Sulfuricellaceae*-associated genomes, except for MAG60. Therefore, the incomplete SOX pathway genes in MAG60 is likely attributed to incomplete binning procedures. The functions of dissimilatory sulfate reduction (*dsr*) pathway genes were inferred by the phylogenetic reconstruction of *dsrA* genes (Supplementary Fig. S7). The reverse *dsr* (r*dsr*) genes, which encode the dissimilatory sulfite reductase that operate in the reverse direction by oxidizing reduced sulfur species [47], were identified as part of the shared core gene set associated within the genus *Sulfuritalea* (Supplementary Fig. S5 and Table S6). Similarly, complete *rdsr* pathway genes were identified in 12 out of 16 *Sulfuricellaceae*-associated genomes (Supplementary Fig. S6 and Table S7). Thus, combining the SIP results, bacteria associated with *Ramlibacter*, *Sulfuricella*, and *Sulfuritalea* are confirmed as SOAsRB based on their genetic potentials of sulfur oxidation and As(V) reduction.

### Phylogenetic diversity and geographical distribution of putative sulfur-oxidizing As(V) reducers

Genome mining was performed to investigate the phylogenetic diversity of putative SOAsRB using bacterial reference genomes from the GTDB database (v207). Potential respiratory As(V)-reducing bacteria were identified based on the presence of *arrAB* genes. Identified *arrA* sequences lacking the conserved motif (R/KGRY) were removed from further analysis [46]. In addition, the presence of gene cluster *arrDE* was required for proposed As(V) reducers. Accordingly, the As(V) reduction potentials were identified in 370 GTDB reference genome assemblies (Fig. 5A), roughly 30% of which (114 genomes) also encoded genes for sulfur oxidation (i.e., *soxB*, reverse *dsr*, and oxidative-type *dsr*) [47–50]. A detailed list of potential As(V)-reducing genomes in the GTDB database is provided in Supplementary Table S8. The *arrA*-containing genomes without sulfur oxidation genes were considered as genomes of non-S-oxidizing As(V) reducers, while the genomes encoding both sulfur oxidation and As(V) reduction genes were considered as genomes of putative SOAsRB. The *arrA* sequences identified in the families *Burkholderiaceae* (35 genomes) and *Rhodocyclaceae* (19 genomes), both of the order *Burkholderiales*, were phylogenetically conserved (Fig. 5A).

Further, quantification of the relative abundances of putative SOAsRB in mine tailings was performed using 17 environmental metagenomes collected from 7 mine tailings sites in Hunan, Guangxi, and Guizhou (Supplementary Table S3 and Fig. 5B). The *arrA* genes were classified as SOAsRB *arrA* genes and non-S-oxidizing *arrA* genes before the quantification process. The presence of As(V) reduction and sulfur oxidation related genes were examined in all available reference genomes of the top 10 SOAsRB genera (Supplementary Table S9). These genera belongs to the family *Burkholderiaceae*, *Rhodocyclaceae*, *Desulfurivibrionaceae*, and other families of *Burkholeriales.* The *arr* genes in these lineages were phylogenetically conserved (Fig. 5A). The sulfur oxidation–related genes were core genetic traits presented in nearly all genomes associated with these genera, while the *arrA* gene was only found in a subset of the genomes. Therefore, if an *arrA* sequence was detected in the reference genome of these genera (i.e. top 10 SOAsRB genera), this *arrA* sequence was considered as an SOAsRB*-*associated *arrA* gene. Subsequently, the *arrA* sequences from both SOAsRB*-*associated *arrA* genes and non–S-oxidizing *arrA* genes were quantified in the environmental metagenomic reads. Putative SOAsRB constituted approximately 87% of the total As(V)-reducing populations in mine tailings, which was significantly higher (*P =* 8*e-*8) compared to non–S-oxidizing As(V)-reducing populations (Fig. 5B). The putative SOAsRB associated with the family *Burkholderiaceae* constituted roughly 65% of the As(V)-reducing populations in mine tailings (Fig. S8), while non–S-oxidizing *Burkholderiacae* only constituted 2% of the overall As(V)-reducing populations. The top 10 most abundant SOAsRB genera constituted roughly 81% of the total As(V)-reducing community and 93% of the SOAsRB community (Fig. 5C). The genus *Rubrivivax* was the most abundant putative SOAsRB identified in the tailing metagenomes, constituting 27.5% of the total As(V)-reducing community. The SOAsRB associated with *Ramlibacter* (9.4%) and *Sulfuritalea* (3.2%) was also among the most abundant As(V)-reducing microorganisms residing in mine tailings.

## Discussion

Arsenate reduction mediated by microorganisms is a key process enhancing As toxicity and mobility, which causes the migration of As from mining regions to the surrounding environment [11]. Compared to well-documented heterotrophic processes [11, 51, 52], chemolithotrophic processes, especially As(V) reduction coupled to S-oxidization, may be more critical and favored by the low TOC and high sulfur conditions in oligotrophic mine tailings [20, 53].The current study suggests that SOAsR is a significant biogeochemical process affecting the fate of As in oligotrophic mine tailing environments.

### Microbial mediated sulfur oxidation coupled to As(V) reduction

The reduction of As(V) to As(III) and thiosulfate oxidation to SO_4_^2−^ was only observed in the As+S treatment (Fig. 1). The co-presence of sulfur oxidation and As(V) reduction only in the cultures amended with As(V) and thiosulfate suggests SOAsR activity. The lack of As(III) and SO_4_^2−^ production in the sterile As+S controls, indicates that SOAsR was primarily microbially mediated. The production of As(III) and SO_4_^2−^ agreed with the theoretical reaction stoichiometry in the ratio of 2:1, providing additional evidence to support microbially mediated sulfur oxidation coupled with As(V) reduction. Thiosulfate is a common substrate utilized by bacteria during sulfur oxidation, and therefore frequently employed for characterization of sulfur-oxidizers [48, 54, 55]. The theoretical Gibbs free energy of thiosulfate oxidation is similar to that of sulfide oxidation, but lower than that of organic substrates as electron donors [22]. Although the energy yield of labile organic substrate oxidation is higher than that of sulfur oxidation [22], the heterotrophic and autotrophic As(V) reduction rates were still comparable in mine tailings investigated in this study (Supplementary Fig. S1), suggesting that the indigenous microbial communities were adapted to the organic substrate–depleted but S-rich geochemical conditions (Supplementary Table S1).

Four geochemical parameters, TOC, WSOC, WSRS, and As concentrations, were positively correlated with SOAsR potential (Supplementary Fig. S2). Many SOAsRB, including members of *Sulfuricella* and *Ramlibacter*, are facultative chemoautotrophs that can use both organic and inorganic substrates [56–58], which could explain the positive correlations between SOAsR potentials and TOC/WSOC concentrations. Nonetheless, compared to organic substrates (TOC/WSOC), reduced sulfur species might be more prevalent electron donors in tailing environments. The WSRS, which is more available for microbial sulfur oxidation, was approximately 8 times higher (molar ratio) than WSOC as measured in this study (Supplementary Table S1). In addition, despite that a majority of sulfur species may be present in the oxidized form (i.e. SO_4_^2−^), the availability of WSRS could be constantly and rapidly replenished due to SO_4_^2−^ reduction in deeper soil layers [59, 60]. In contrast, the tailings are oligotrophic with low TOC (~1%) compared to regular soils (between 1% and 6%) [61], whereas WSOC, which is the readily bioavailable fraction of TOC, only represents a minor fraction (~ 0.4%–3.4%) of TOC in the studied tailings (Supplementary Table S1) [62, 63]. Indeed, microorganisms have been shown to adapt to harsh oligotrophic conditions and use sulfur as a primary energy source in diverse ecosystems [20, 64, 65]. Therefore, combining the geochemical analysis and the rate measurements, we propose that reduced sulfur species, rather than organic substrates, are the primary electron donors that mediate As(V) reduction in mine tailings.

### Identification of SOAsRB

Significant changes in the microbial community compositions were observed during the SOAsR incubations (Supplementary Fig. S3A and S3B) with several bacterial taxa found in high abundances (Supplementary Fig. S3C). Among these dominant populations, members of multiple genera, such as *Geobacter*, *Desulfitobacterium*, and *Aquabacterium*, were proposed as potential As(V) reducers through our genome mining analysis (Fig. 5). Although members of *Geobacter* and *Desulfitobacterium* spp. have been confirmed as As(V) reducers [45, 66], S oxidation has not previously been reported in these genera. This observation suggested that in addition to SOAsR, heterotrophic As(V) reduction might have occurred during the incubation.

To pinpoint SOAsRB, DNA-SIP was performed as only the active SOAsRB were expected to assimilate ^13^C-labeled bicarbonate [67]. A combination of metagenomic binning and pangenome analysis was included as a further confirmation of the genetic capacities for identified SOAsRB. Accordingly, bacteria associated with the genera *Sulfuricella*, *Sulfuritalea*, and *Ramlibacter* were confirmed as SOAsRB by DNA-SIP. Further, MAGs associated with *Sulfuricella* and *Ramlibacter* contained *arr* sequences. The *arr* sequences identified in these MAGs all encoded the conserved motif (R/KGRY), which distinguish them from the closely-related anaerobic As(III) oxidase *arx* gene [46]. All *arrAB* genes in these MAGs were flanked by *arrDE* genes, which encode a molecular chaperone TorD-like protein and a putative 4Fe-4S protein, respectively, the same as the previously reported gene arrangement in the As(V)-reducing *Geobacter* sp. OR-1 [45]. Combined, this suggests that the identified SOAsRB encode functioning As(V)-reducing Arr.

While the *arr* genes were not identified, the respiratory As(V) reduction capacity by *Sulfuritalea*-associated MAGs was predicted. Although it has been suggested that As(V) reduction capacity was not shared among closely related strains [68], our phylogenetic analysis indicates that the *arr* genes are conserved within certain lineages, including members of the family *Rhodocyclaceae*, to which *Sulfuritalea* belongs (Fig. 5A). Accordingly, the *Sulfuritalea*-related *arrA* genes clustered together in the phylogenetic reconstruction. Given the divergent geographical origins of these genomes (Ohio, California, Puerto Rico, and Japan; Supplementary Table S6), the *arrA* gene could be vertically transmitted within a subset of *Sulfuritalea* populations rather than acquired through horizontal gene transfer (HGT) [68]. Moreover, multiple *arrA* sequences, along with the ORFs flanking them, were identified in assembled SIP–metagenome contigs and these sequences were closely related to genes retrieved from *Sulfuritalea*-associated genomes, suggesting that the lack of *arr* genes in *Sulfuritalea*-associated MAGs was caused by incomplete binning.

The As(V) reduction potential might be vertically transmitted within *Ramlibacter* populations, as the *arrA* sequences of the *Ramlibacter*-associated MAGs clustered together with other *arrA* genes retrieved from members of the family *Burkholderiaceae*, to which the *Ramlibacter* belongs (Fig. 5A). In contrast, the *Sulfuricella-*associated MAG60 was the only *arrA*-encoding genome within the available genomes of the family *Sulfuricellaceae*. Meanwhile, its *arrA* gene shared high sequence identity with the *arrA* genes retrieved from members of *Desulfobacterota*. This finding suggests that the *Sulfuricellaceae* MAG60 may have acquired the As(V) reduction capacity through HGT. Indeed, the *arrA* gene is frequently transferred among bacterial populations through HGTs [68], which have been frequently found in As-contaminated sites as a response to elevated environmental (As) stress [69].

The sulfur oxidization–related genes were identified as core gene clusters for all three identified SOAsR populations (Supplementary Fig. S5–S6 and Supplementary Table S8). The co-presence of both sulfur oxidization and As(V) reduction genes in retrieved *Sulfuricella-* and *Ramlibacter*-associated MAGs suggested their potential for coupling sulfur oxidation with As(V) reduction. These three genera are known sulfur oxidizers that frequently thrive in environments with active sulfur cycles, such as salt lakes, groundwater, and mine tailings [70–74]. The capability for As(V) reduction has been confirmed in a *Sulfuritalea* isolate [75]. Members of the genus *Ramlibacter* were reported as dominant members in heterotrophic As(V) reducing enrichment cultures [76, 77]. While As(V) reduction has not been reported for *Sulfuricella* species, some strains of this genus have been isolated from contaminated environments containing high concentrations of As [78–80].

### Phylogenetic diversity of putative SOAsRB and their distribution in tailings

Mining of the GTDB reference genomes provided additional information on the phylogenetic diversity of putative SOAsRB and an estimation of the potential significance of SOAsR in various tailing environments. Genome mining suggested that diverse microbial populations encode the essential genes (i.e. both sulfur oxidation and As(V) reduction genes) for SOAsR and were identified as putative SOAsRB (Fig. 5A and Supplementary Fig. S7). For example, the *Burkholderiaceae* and *Rhodocyclaceae* were two of the microbial families that contained most putative SOAsRB. The families within the order *Desulfobulbales* (i.e. *Desulfobulbaceae*, *Desulfocapsaceae*, and *Desulfurivibrionaceae*) were another major microbial populations harboring putative SOAsRB. Several pure isolates of *Desulfobulbales* can perform sulfur oxidation coupled to As(V)/Sb(V) reduction. In fact, strain MLMS-1, a member of the family *Desulfurivibrionaceae* isolated from Mono Lake, USA, was the first reported as a SOAsRB [22]. Moreover, an *arrA*-containing *Desulfurivibrio*, another member of *Desulfurivibrionaceae*, is capable of performing sulfur oxidation coupled with the reduction of Sb(V), an As(V) analog [48]. *H. silvermanii* strain SLAS-1 (RS_GCF_900103135.1), a strain with confirmed SOAsR activity [23], was predicted to be an As(V) reducer through our genome mining, even though major sulfur oxidation pathway genes (SOX or *dsr*) were not identified in the genome. Together, these observations suggest that a wide diversity of bacterial populations have the metabolic potentials to perform SOAsR.

The meta-analysis of metagenomes of As-rich mine tailings across southwestern China revealed high abundances of putative SOAsRB in the total As(V)- reducing community, as the SOAsRB-associated *arrA* genes dominated the overall *arrA* genes retrieved from tailing metagenomes. This suggests that SOAsR may play an important role for biogeochemical As cycling in mine tailings. A substantial portion of *arrA* sequences in tailing metagenomes were affiliated with the SOAsRB identified by DNA-SIP or genome mining (Fig. 3, 4, and 5B). For instance, members of *Rubrivivax* were identified as the most abundant putative SOAsRB in mine tailing metagenomes with relative abundances greater than 20%. In addition, *Ramlibacter* and *Sulfuritalea* identified as SOAsRB by SIP were among the most abundant As(V)-reducing populations in mine tailings. While these SOAsRB might be facultative chemolithoautotrophs [56], the low availability of organic carbon in tailings prompt these SOAsRB to use sulfur as a primary electron donor for As(V) reduction, making SOAsR a dominant process for As(V) reduction.

## Conclusions

Unique geochemical conditions (i.e. low organic carbon availability and high sulfur content) have been shown to promote microbial S-oxidization [20], which may further be coupled to As(V) reduction in oligotrophic As-contaminated environments. This study suggests that biological SOAsR can be a pivotal process in regulating As speciation in tailing environments. The As(V) reduction rates were comparable between cultures using equal concentrations of thiosulfate or organic carbon as the sole electron donor. Phylogenetically diverse lineages of SOAsR were affirmed or suggested by a combination of DNA-SIP, metagenome, and genome mining analysis. Further, these (putative) SOAsR dominated the community of As(V) reducing bacteria in tailing metagenomes, as observation that agreed with the activity measurements of heterotrophic As(V) reduction and chemolithotrophic As(V) reduction, indicating that the indigenous microbial community was adapted to using sulfur as an electron donor in oligotrophic mine tailings. These findings reveal the importance of microbial sulfur oxidation to As biogeochemical cycling in oligotrophic As-contaminated sites, which may be employed to facilitate future remediation of mine tailings.

## Supplementary Material

Supplementary_Info_wrae110

Fig_S7_wrae110

SOAsR_SI-Tables_wrae110

## Data Availability

All sequences generated in the current study was deposited to the NCBI SRA database under the bioproject PRJNA989741. The accessions for metagenomes are provided in Supplementary Table S3. The data of SOAsR incubations is available in Figshare: 10.6084/m9.figshare.25189016. Correspondence and requests for materials should be addressed to Weimin Sun.
