# Supplementary material for: Microbially mediated sulfur oxidation coupled with arsenate reduction within oligotrophic mining–impacted habitats
Source: ISME J. 2024 Jun 20;18(1):wrae110. doi: 10.1093/ismejo/wrae110 (PMC11283718; doi:10.1093/ismejo/wrae110)
Supplement: Supplementary_Info_wrae110 [file supplementary_info_wrae110.docx]

**Supplementary Information**

**Microbially mediated sulfur oxidation coupled with arsenate reduction within oligotrophic mining-impacted habitats**

Xiaoxu Sun ^1,2#^, Qizhi Chen ^1,3#^, Max M. Häggblom ^4^, Guoqiang Liu ^3^, Tianle Kong ^1,5^, Duanyi Huang ^1,6^, Zhenyu Chen ^1,7^, Fangbai Li ^1,2^, Baoqin Li ^1,2^, and Weimin Sun ^1,2^*

^1^ National-Regional Joint Engineering Research Center for Soil Pollution Control and Remediation in South China, Guangdong Key Laboratory of Integrated Agro-environmental Pollution Control and Management, Institute of Eco-environmental and Soil Sciences, Guangdong Academy of Sciences, Guangzhou 510650, China

^2^ Guangdong-Hong Kong-Macao Joint Laboratory for Environmental Pollution and Control，Guangzhou Institute of Geochemistry, Chinese Academy of Sciences, Guangzhou 510640, China

^3^ Guangdong Key Laboratory of Environmental Pollution and Health, School of Environment, Jinan University, Guangzhou 511443, China

^4^ Department of Biochemistry and Microbiology, Rutgers University, ﻿New Brunswick, NJ 08901, USA

^5^ College of Environmental Science and Engineering, Donghua University, Shanghai 201620, China

^6^ College of Environmental Science and Engineering, Hunan University, Changsha 410082, China

^7^ School of Environment, Key Laboratory of Yellow River and Huai River Water Environment and Pollution Control, Ministry of Education, Henan Normal University, Xinxiang 453007, China

# These two authors contributed equally to the manuscript.

*Correspondence to:

Dr. Weimin Sun

808 Tianyuan Road, Guangzhou, Guangdong, China

Phone: 86-020-87024633

Fax: 86-020-87024123

Email: wmsun@soil.gd.cn

### Supplementary Methods 1. Geochemical analysis

The tailing samples were first freeze-dried and then passed through a 100-mesh screen. For pH measurement, the 1 g freeze-dried soil samples were mixed with 10 ml distilled water and then measured with an HQ30d pH meter (Hach, CO, USA).

The total organic carbon (TOC) was analyzed using 1 g of dried sample on a Shimadzu TOC analyzer with a solid sample module 5000A (Shimadzu, Japan). Water-soluble organic carbon (WSOC) was analyzed by first thoroughly mixing 2 g tailing with 10 ml DI water. The supernatant was then passed through a 0.45 μm filter followed by acidification to pH =2 with HCl and aeration with O_2_ for 11 min. The samples were then analyzed on the TOC analyzer.

For total nitrogen (TN) analysis, 0.1 g of tailing sample was mixed with 10 ml DI water and 5 ml sodium persulfate. The mixture was digested at 120 ℃ for 1 h. The mixture was then diluted to 25 ml and analyzed on an Ion Chromatograph (ICS-40, Dionex, CA, USA).

For total sulfur (TS) measurement, 0.1 g tailing was mixed with 10 ml H_2_O_2_ and 50 uL formic acid for 2 hrs followed by adding 10 ml H_2_O_2_ for additional 2 hr. The final volume was diluted to 20 ml and then passed through a 0.45 μm filter. The solution was analyzed on an Ion Chromatograph (ICS-40, Dionex, CA, USA). Water-soluble SO_4_^2-^ (WSS) was analyzed by first thoroughly mixing 2 g of tailing sample with 10 ml DI water and the supernatant analyzed on the IC. For Water-soluble reduced sulfur (WSRS), the supernatant was further mixed with H_2_O_2_ (25:1 v/v) and exposed to UV light for 24 h, then the supernatant analyzed on the IC.

The total Sb and As concentrations were analyzed on an LC-AFS (Haiguang, Beijing, China) after digestion with ﻿5:1 (v/v) HNO_3_ and HF. For all incubations, the changes in As and sulfur species were monitored in the supernatant using LC-AFS, and IC, respectively.

### Supplementary Methods 2. Activity and SOAsR incubation setup

The activity characterization of the S-oxidizing mediated As(V) reduction (SOAsR) in the mine tailing was performed using enrichment cultures (i.e., activity analysis cultures). A total of 4 treatments (5 replicates for each treatment) were prepared: (i) As+S treatment contained both 0.5 mM As(V) (sodium arsenate 97%, Guangzhou Chemical Agents, Guangdong, China) and 0.5 mM thiosulfate (potassium thiosulfate 99%, Macklin biochemical, Shang, China); (ii) As-only treatment contained 0.5 mM As(V); (iii) S-only treatment contained 0.5 mM thiosulfate; and (iv) Sterile As+S treatment inoculated with sterilized tailing sample and 0.5 mM As(V) and 0.5 mM thiosulfate. The sterilized tailing sample was prepared using gamma-radiation at 25 kGy for 10 min. For better estimation of stoichiometry of the reaction, the As+S cultures were enriched by transferring 20% of the total volume to the next generation enrichment cultures. The cultures were transferred twice to reduce soil particles, which may cause As adsorption.

Additional incubations were performed to compare the heterotrophic As(V) reduction rates using an organic substrate (heterotrophic, 1 mM sodium acetate) and chemotrophic As(V) reduction rates using a reduced sulfur substrate (1 mM thiosulfate). Controls without electron donor addition was included to demonstrate the background As(V) reduction rates.

The relationship between SOAsR potential and geochemical parameters were tested using enrichment cultures. Tailing samples collected from 6 different tailing sites (FK, FK2, CSWK, HJ, TXX, and XKS) were employed as inoculum. The sulfur-oxidizing As(V) reducing cultures were prepared with 1 g of tailing samples, 1 mM of thiosulfate, and 0.5 mM As(V). The correlations between geochemical parameters and As(V) reduction rates were analyzed using Pearson correlation in ‘stats’ package in R. The changes in microbial community compositions were monitored during the incubation. Triplicate samples were sacrificed for DNA extraction and amplicon sequencing at four different time points (Day 0, 10, 20, and 30).

### Supplementary Method 3. Stable isotope probing culture setup and sample processing

Stable isotope probing (SIP) cultures were prepared for identification of putative SOAsRBs. The setup of the SIP cultures was the same as for the activity analysis cultures but with different carbon isotope substrates. Either ^13^C-labeled bicarbonate (99%, Cambridge Isotope Laboratories, MA, USA) or ^12^C-unlabeled bicarbonate was provided. Four treatments (triplicate microcosms for each treatment) were prepared (i.e. ^13^C-As+S amended with ^13^C-labeled bicarbonate, As(V), and thiosulfate; ^12^C-As+S amended with ^12^C-labeled bicarbonate, As(V), and thiosulfate; ^13^C-As amended with ^13^C-labeled bicarbonate and As(V); and ^13^C-S amended with ^13^C-labeled bicarbonate and thiosulfate). The cultures were incubated for 20 days and then sacrificed for subsequent analyses. Genomic DNA was extracted from samples using the DNeasy Powersoil Kit (Qiagen, Dresden, Germany) following the manufacturer’s protocol. Equal amounts of DNA (500 ng) from each sample were mixed with CsCl solution to a final density of 1.714 g/mL and ultracentrifuged in a heat-sealable OpitSeal Polypropylene tube (Beckman Coulter, CA, USA) at 408,500 g for 40 h (Beckman Coulter, CA, USA). The isopycnic fractions were recovered using a fraction recovery system (Beckman Coulter, CA, USA) and the buoyant density of each fraction was measured using a refractometer (Agato, Tokyo, Japan).

### Supplementary Method 4. Bioinformatic analysis

The time-series samples from the SOAsR incubations and a subset of SIP fractions (**Table S2**) were selected for amplicon sequencing based on the qPCR results. The primer set 515F/806R targeting the V4 hypervariable region of the 16S rRNA gene was employed. The amplicons were sequenced on an Illumina NovaSeq at Personal Biotechnology, Shanghai, China. The raw paired-end reads were processed in QIIME2 [1]. Briefly, the raw sequences were trimmed, filtered for quality control, and the chimeric reads were removed. The final clean reads were merged and clustered into amplicon sequencing variants (ASVs) with DADA2 [2]. The taxonomy of the ASVs were assigned against the SILVA 138 database [3]. The ASV abundance table and taxonomy table were then imported as a phyloseq object in R [4] and visualized using ggplot2 [5].

The unfractionated total DNA from the ^13^C-As+S treatment was pooled and used for metagenomic sequencing on an Illumina NovaSeq at the Personal Biotechnology, Shanghai, China. The raw reads were trimmed for quality control purposes using Trimmomatic [6]. The clean reads were assembled using Metaspades with k-list 21 to 121, step = 10 [7]. Metagenome reads were binned into metagenome assembled genomes (MAGs) using metabat2 [8], maxbin2 [9], and VAMB [10], respectively. The MAGs were then refined using Metawrap [11] and dereplicated using dRep [12]. The quality of MAGs was estimated using CheckM with the lineage_wf workflow [13]. The taxonomy of the MAGs was assigned using GTDB-tk [14] against the GTDB database (version r207) [15]. The open reading frames (ORFs) were predicted using Prodigal [16]. The functional annotation of the ORFs were analyzed using kofamscan against the KEGG database and the completeness of metabolic pathways were estimated with KEGGdecoder [17, 18]. Pangenomic analysis of potential sulfur-oxidizing As(V) reducing bacteria (SOAsRB) were performed using reference genomes retrieved from GTDB r207 in Anvi’o using the pangenomic workflow [19]. The average nucleotide identity (ANI) among the genomes were calculated using fastANI [20].

Phylogenetic diversity of potential SOAsRB was estimated using reference genomes from the GTDB v207 database. The amino acid sequences were searched against *arrA* sequences using HMMER3 with threshold > 1158 [21]. The sulfur metabolizing genes *soxB* and *dsrA* were searched against their respective databases using recommended thresholds of 412 and 304, respectively [17]. The retrieved amino acid sequences of the functional genes were aligned using Muscle and then the phylogenetic tree was constructed using RaxML (model = LG + G8, bootstrap = 200) [22, 23]. A total of 17 metagenomes from 7 mine tailings were employed for quantification of the abundance SOAsRs compared to that of Non-S-oxidizing As(V) reducers (**Table S3**). The *arrA* sequences retrieved from assembled MAGs and reference genomes were quantified in the metagenomes using Salmon (flag: --meta –validateMapping –hardFilter –mimicStrictBT2) [24].

### References

1. Bolyen E, Rideout JR, Dillon MR, Bokulich NA, Abnet CC, Al-Ghalith GA, et al. Reproducible, interactive, scalable and extensible microbiome data science using QIIME 2. *Nat Biotechnol* 2019; **37**: 852–857.

2. Callahan BJ, Mcmurdie PJ, Rosen MJ, Han AW, Johnson AJA, Holmes SP. Dada2: High resolution sample inference from Illumina amplicon data. *Nat Methods* 2016; **13**: 581–583.

3. Quast C, Pruesse E, Yilmaz P, Gerken J, Schweer T, Yarza P, et al. The SILVA ribosomal RNA gene database project: Improved data processing and web-based tools. *Nucleic Acids Res* 2013; **41**.

4. McMurdie PJ, Holmes S. Phyloseq: An R Package for Reproducible Interactive Analysis and Graphics of Microbiome Census Data. *PLoS One* 2013; **8**: e61217.

5. Wickham H. ggplot2: Elegant graphics for data analysis. *Wiley Interdisciplinary Reviews: Computional Statistics* 2011; **3**: 180–185.

6. Bolger AM, Lohse M, Usadel B. Trimmomatic: A flexible trimmer for Illumina sequence data. *Bioinformatics* 2014; **30**: 2114–2120.

7. Bankevich A, Nurk S, Antipov D, Gurevich AA, Dvorkin M, Kulikov AS, et al. SPAdes: A New Genome Assembly Algorithm and Its Applications to Single-Cell Sequencing. *Journal of Computational Biology* 2012.

8. Kang DD, Li F, Kirton E, Thomas A, Egan R, An H, et al. MetaBAT 2: An adaptive binning algorithm for robust and efficient genome reconstruction from metagenome assemblies. *PeerJ* 2019; **2019**: 1–13.

9. Wu YW, Simmons BA, Singer SW. MaxBin 2.0: An automated binning algorithm to recover genomes from multiple metagenomic datasets. *Bioinformatics* 2016; **32**: 605–607.

10. Nissen JN, Johansen J, Allesøe RL, Sønderby CK, Armenteros JJA, Grønbech CH, et al. Improved metagenome binning and assembly using deep variational autoencoders. *Nat Biotechnol* 2021; **39**: 555–560.

11. Uritskiy G V., Diruggiero J, Taylor J. MetaWRAP - A flexible pipeline for genome-resolved metagenomic data analysis 08 Information and Computing Sciences 0803 Computer Software 08 Information and Computing Sciences 0806 Information Systems. *Microbiome* 2018; **6**: 1–13.

12. Olm MR, Brown CT, Brooks B, Banfield JF. DRep: A tool for fast and accurate genomic comparisons that enables improved genome recovery from metagenomes through de-replication. *ISME Journal* 2017; **11**: 2864–2868.

13. Parks DH, Imelfort M, Skennerton CT, Hugenholtz P, Tyson GW. CheckM: assessing the quality of microbial genomes recovered from isolates, single cells, and metagenomes. *Genome Res* 2015; **25**: 1043–55.

14. Chaumeil PA, Mussig AJ, Hugenholtz P, Parks DH. GTDB-Tk: A toolkit to classify genomes with the genome taxonomy database. *Bioinformatics* 2020; **36**: 1925–1927.

15. Parks DH, Chuvochina M, Rinke C, Mussig AJ, Chaumeil P-A, Hugenholtz P. GTDB: an ongoing census of bacterial and archaeal diversity through a phylogenetically consistent, rank normalized and complete genome-based taxonomy. *Nucleic Acids Res* 2022; **50**: D785–D794.

16. Hyatt D, Chen G-L, Locascio PF, Land ML, Larimer FW, Hauser LJ. Prodigal: prokaryotic gene recognition and translation initiation site identification. *BMC Bioinformatics* 2010; **11**: 119.

17. Aramaki T, Blanc-Mathieu R, Endo H, Ohkubo K, Kanehisa M, Goto S, et al. KofamKOALA: KEGG Ortholog assignment based on profile HMM and adaptive score threshold. *Bioinformatics* 2020; **36**: 2251–2252.

18. Graham ED, Heidelberg JF, Tully BJ. Potential for primary productivity in a globally-distributed bacterial phototroph. *ISME Journal* 2018; **12**: 1861–1866.

19. Eren AM, Esen OC, Quince C, Vineis JH, Morrison HG, Sogin ML, et al. Anvi’o: An advanced analysis and visualization platformfor ’omics data. *PeerJ* 2015; **2015**: 1–29.

20. Jain C, Rodriguez-R LM, Phillippy AM, Konstantinidis KT, Aluru S. High throughput ANI analysis of 90K prokaryotic genomes reveals clear species boundaries. *Nat Commun* 2018; **9**: 1–8.

21. Mistry J, Finn RD, Eddy SR, Bateman A, Punta M. Challenges in homology search: HMMER3 and convergent evolution of coiled-coil regions. *Nucleic Acids Res* 2013; **41**.

22. Edgar RC. MUSCLE: A multiple sequence alignment method with reduced time and space complexity. *BMC Bioinformatics* 2004; **5**: 1–19.

23. Kozlov AM, Darriba D, Flouri T, Morel B, Stamatakis A. RAxML-NG: A fast, scalable and user-friendly tool for maximum likelihood phylogenetic inference. *Bioinformatics* 2019; **35**: 4453–4455.

24. Patro R, Duggal G, Love MI, Irizarry RA, Kingsford C. Salmon: fast and bias-aware quantification of transcript expression using dual-phase inference. *Nat Methods* 2017; **14**: 417–419.

**Figure S1.** As(V) reduction rate under heterotrophic and chemoautotrophic conditions. Thiosulfate (S+As) and acetate (Acetate +As) were employed as the sole electron donor in the chemoautotrophic and heterotrophic treatments, respectively. Controls without thiosulfate or acetate addition were also included. The error bars indicate the standard deviation of 5 replicates.

**Figure S2.** A) Sulfur-oxidizing As(V) reduction (SOAsR) potentials, as proxied by the As(V) reduction rates, in 6 different tailing samples. B) Correlations between the SOAsR potential and geochemical parameters. Numbers indicated the Pearson correlation R value. Crossed values indicate the statistical significance *p >* 0.05. Site abbreviations: CSWK- ChashanWeikuang, Pb/Zn/Sb mine tailing in Nandan, Guangxi; FK/FK2- Fankou Pb/Zn mine tailings in Guangdong; HJ- Huangjia Sb mine tailing in Hunan; TXX- Tangxi Pb/Zn mine tailing in Hunan; and XKS Sb mine tailing in Hunan.


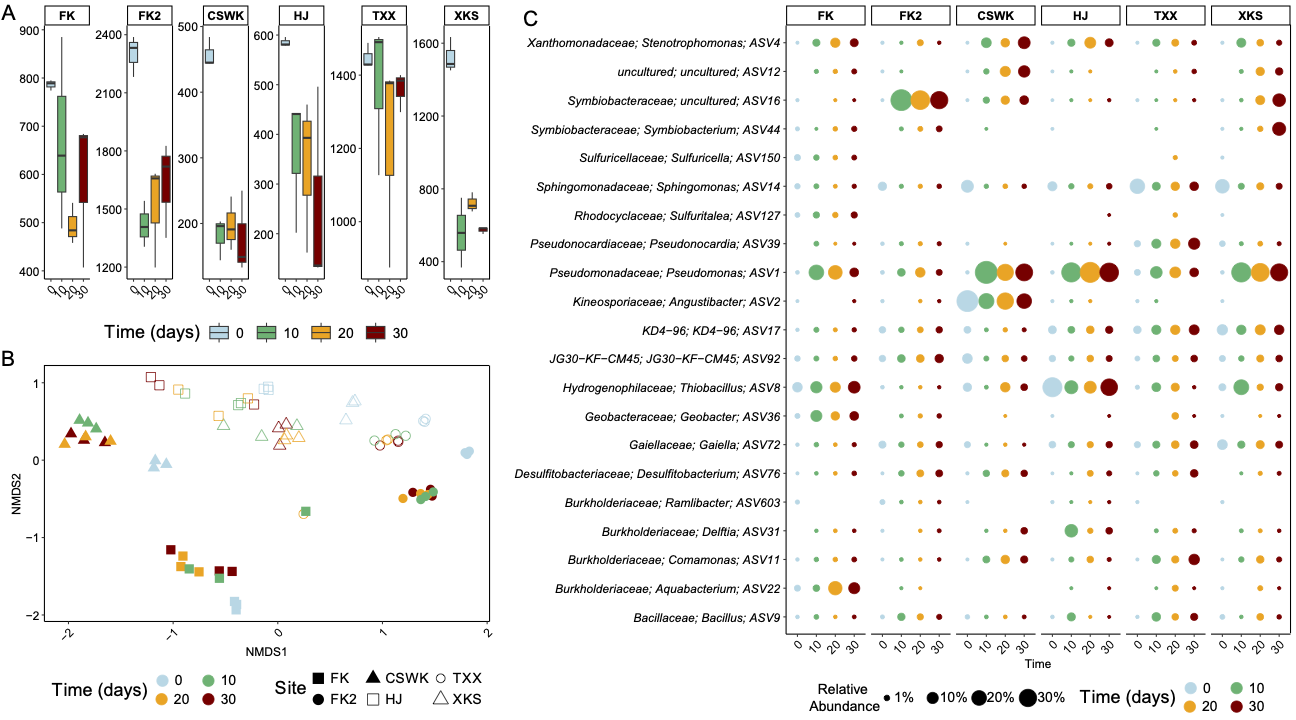


**Figure S3.** Changes in microbial community compositions during SOAsR incubations. A) alpha diversity in the measurement of Observed species. B). NMDS plot of Bray-Curtis distance. C) dominant microbial populations at the genus level. Site abbreviations: CSWK- ChashanWeikuang, Pb/Zn/Sb mine tailing in Nandan, Guangxi; FK/FK2- Fankou Pb/Zn mine tailings in Guangdong; HJ- Huangjia Sb mine tailing in Hunan; TXX- Tangxi Pb/Zn mine tailing in Hunan; and XKS Sb mine tailing in Hunan.


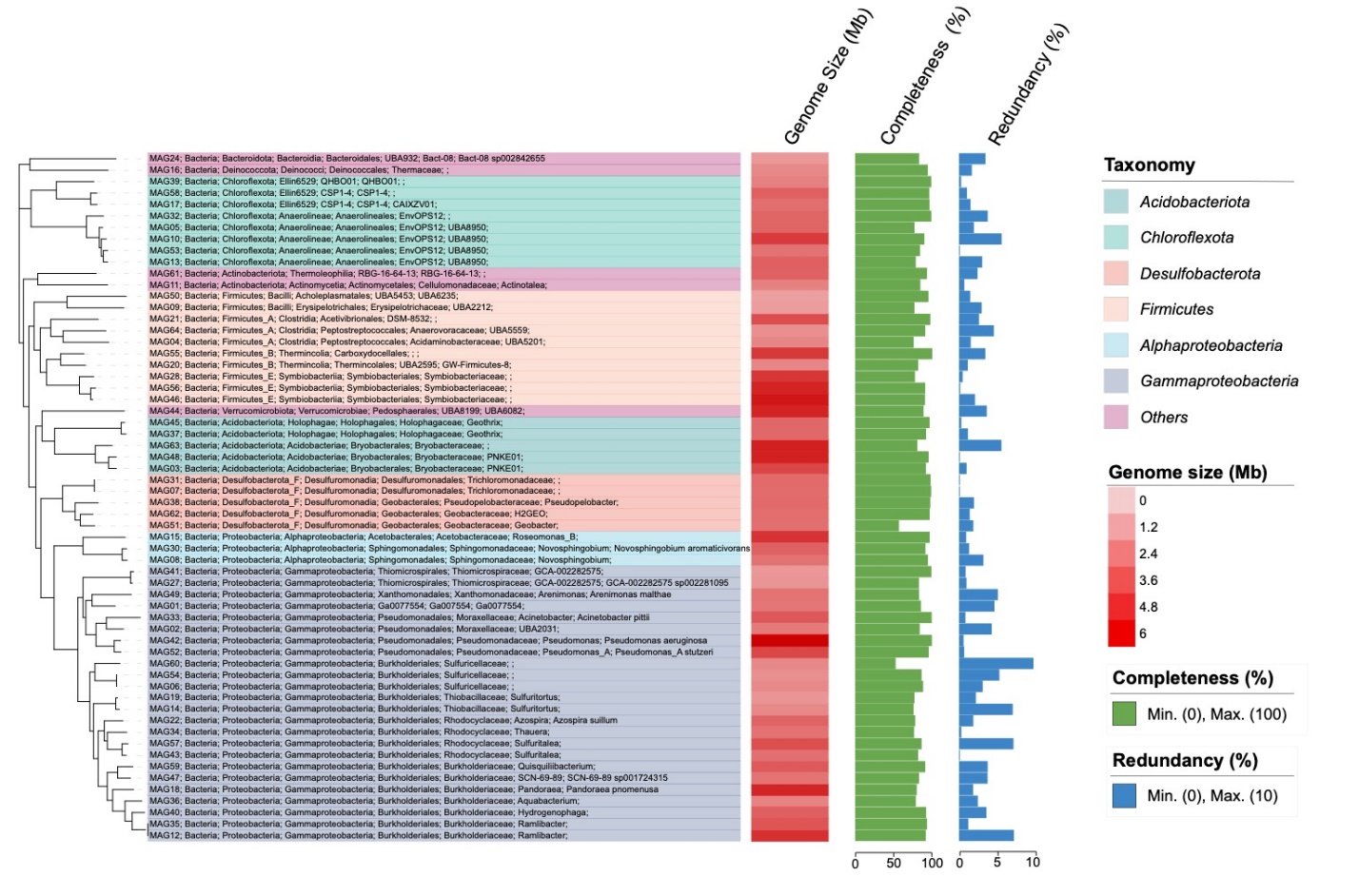


**Figure S4.** Phylogenetic reconstruction of metagenome assembled genomes (MAGs) from SIP metagenomes. Details are provided in Supplementary Table S5.


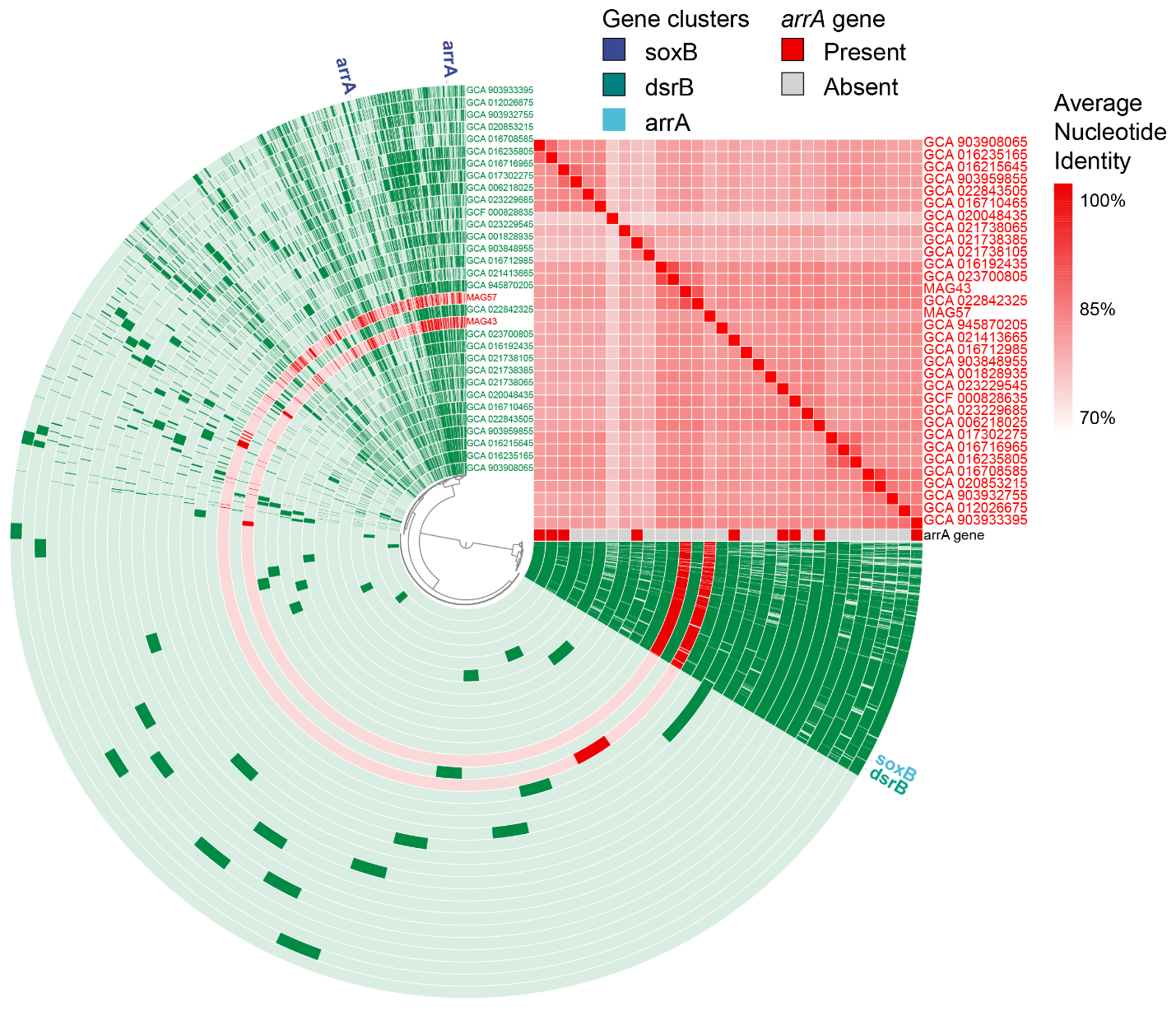


**Figure S5.** Pangenome analysis of *Sulfuritalea* genomes. Red layers represent MAGs retrieved from the current study; green layers represent GTDB reference genomes. Dark colors (both dark red and green) represent genes encoded in the genome. Details are provided in Supplementary Table S6.


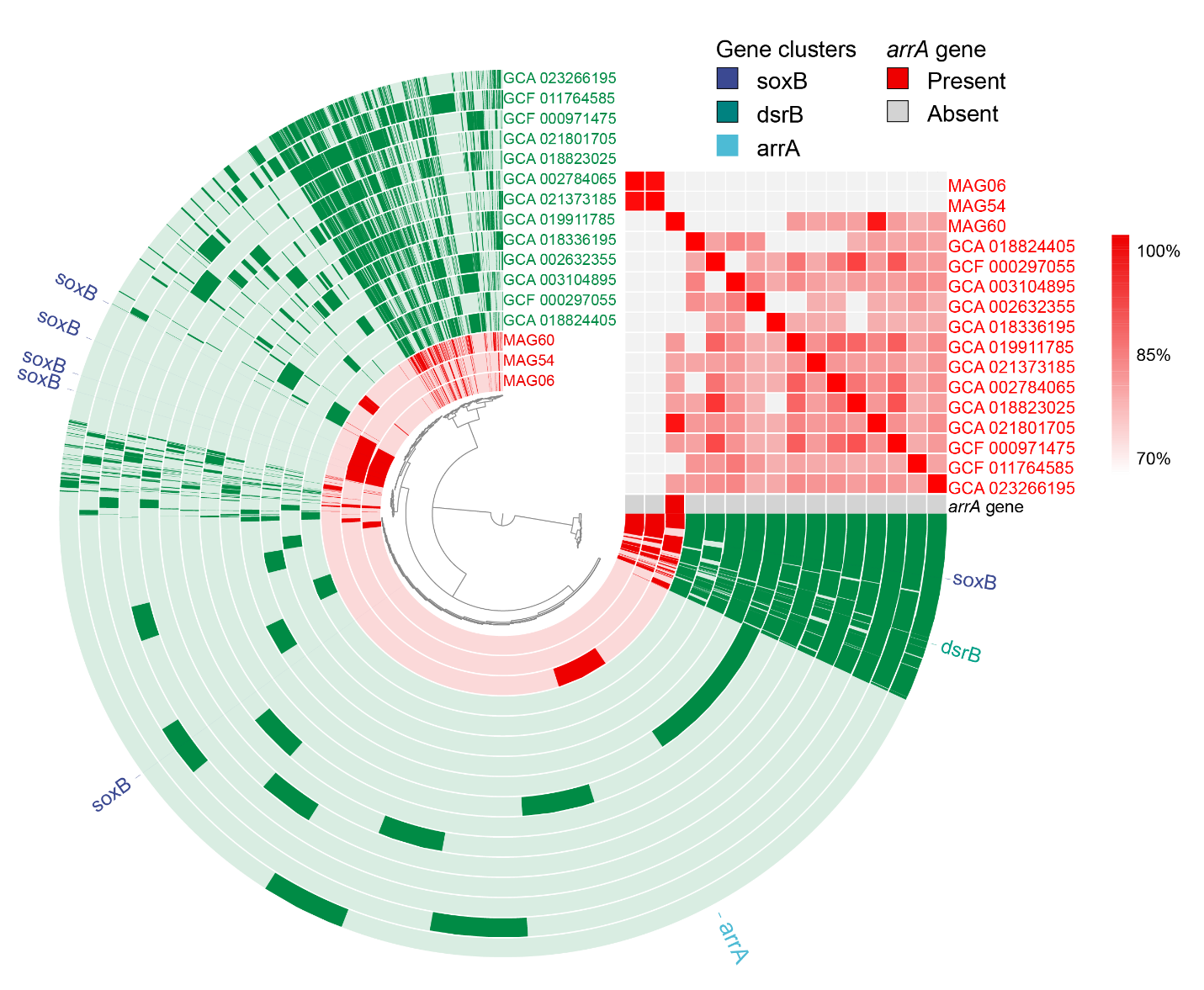


**Figure S6.** Pangenome analysis of *Sulfuricellaceae* genomes. Red layers represent MAGs retrieved from the current study; green layers represent GTDB reference genomes. Dark colors (both dark red and green) represent genes encoded in the genome. Details are provided in Supplementary Table S7.

Figure S7 is provided as an individual supplementary file.

**Figure S8.** Relative abundance of the dominant As(V) reducing microbial families in tailing metagenomes.
