# Supplementary material for: Microbially mediated sulfur oxidation coupled with arsenate reduction within oligotrophic mining–impacted habitats
Source: ISME J. 2024 Jun 20;18(1):wrae110. doi: 10.1093/ismejo/wrae110 (PMC11283718; doi:10.1093/ismejo/wrae110)
Supplement: Fig_S7_wrae110 [file fig_s7_wrae110.pdf]

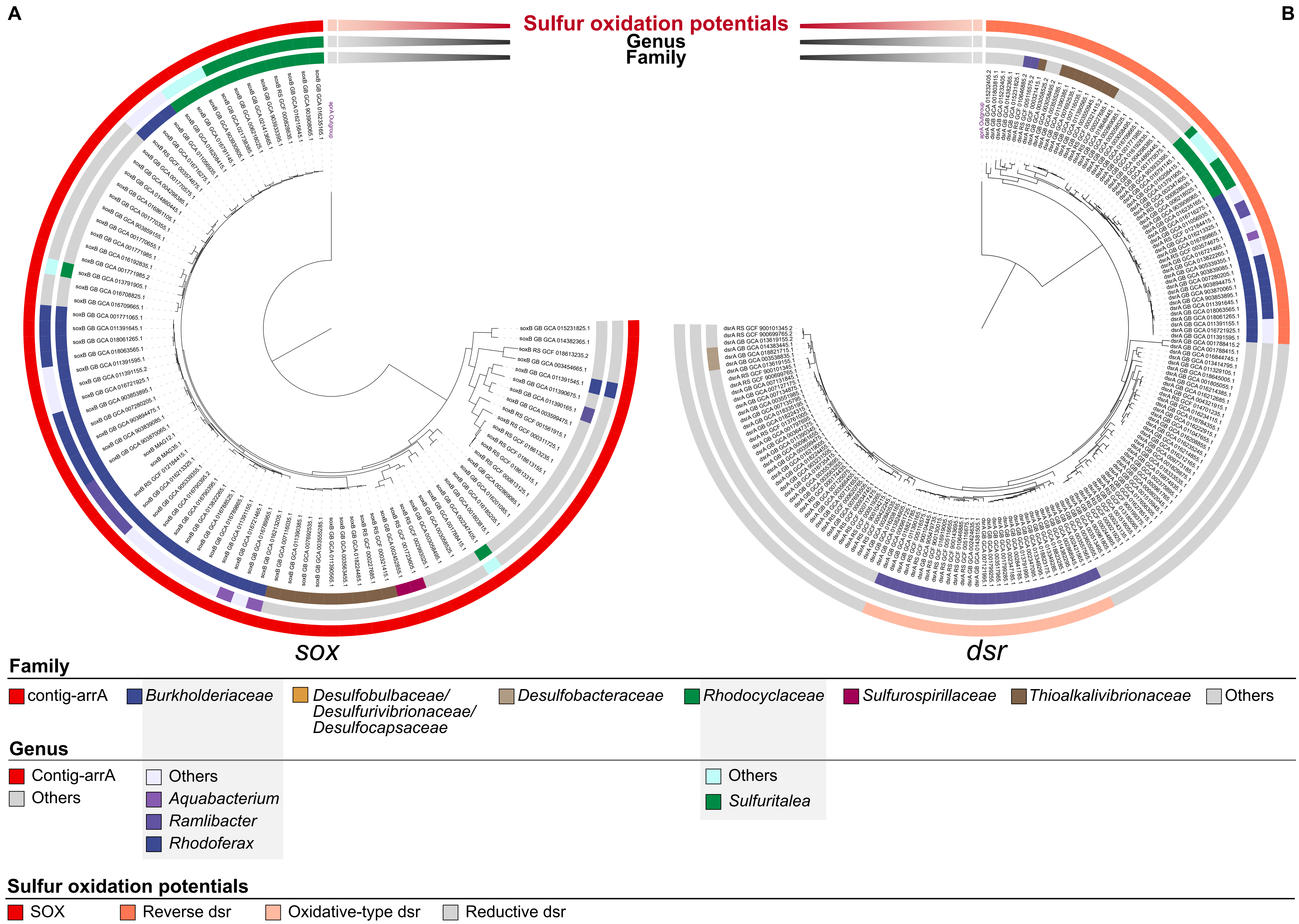

Figure S7. Phylogenetic tree of the soxB (A) and dsrA (B) gene from arrA-containing MAGs and GTDB reference genomes. An aprA gene was introduced as the outgroup in both trees.
